# Transient Receptor Potential Ankyrin 1 Channels in Inflammation and Cancer: Accelerators or Brakes?

**DOI:** 10.1093/function/zqac049

**Published:** 2022-09-17

**Authors:** Shuang Peng

**Affiliations:** School of Sport and Health Sciences, Guangzhou Sport University, Guangzhou 510500, China; Key Laboratory of Sports Technique, Tactics and Physical Function of General Administration of Sport of China, Scientific Research Center, Guangzhou Sport University, Guangzhou 510500, China

**Keywords:** TRPA1, Ca^2+^ signaling, pancreatic stellate cells, pancreatic fibrosis, alcoholic pancreatitis

The TRP channel TRPA1, also known as the transient receptor potential ankyrin 1 channel or the Wasabi receptor, is regarded as an important mediator of the inflammatory actions of environmental irritants.^1,2^ Although the channel has been primarily studied in nerves, it is present in many different tissues.^1^ It is a relatively nonselective cation channel with a significant permeability for Ca^2+^ and opening of the channel will therefore mediate Ca^2+^ influx.^1^

It has been shown that TRPA1 can be activated by a number of noxious chemical agents as well as by the inflammatory peptide bradykinin (BK).^3^ BK has for a long time been implicated in the inflammatory disease acute pancreatitis^4^ and since TRPA1 is expressed widely in the gastro-intestinal tract^1^, it would be of interest to investigate the possible role of this channel in the pathophysiology of pancreatitis. It is now well established that BK elicits Ca^2+^ signals in the pancreatic stellate cells, but not in the quantitatively dominant acinar cells.^5^ In this context, it is interesting that Kusiak et al.^6^ have now shown that the TRPA1 channel is expressed in human pancreatic stellate cells and mediates Ca^2+^ signals of pathophysiological importance.

The stellate cells form a relatively small component of the normal exocrine pancreas, where they exist in a so-called quiescent state.^7^ In spite of this designation, intracellular Ca^2+^ signals are elicited by concentrations of BK that are only very slightly above the normal resting plasma level.^5^ It is well established that BK plasma levels are elevated in acute pancreatitis and that at such concentrations, BK elicits vigorous Ca^2+^ signal generation in the stellate cells.^4,5^ It is also known that in pancreatitis, the stellate cells can become transformed into an activated state and that in chronic pancreatitis, these activated stellate cells become a large component of the exocrine pancreas, contributing to the production of an inflammatory microenvironment that is potentially cancer promoting.^7^

Kusiak et al.^6^ now present data that provide a plausible explanation for the well-established fact that whereas alcohol-related acute pancreatitis often develops into chronic pancreatitis, this is not the case for bile-related acute pancreatitis.^5,7,8^ Bile acids are capable of inducing acute pancreatitis elicit large and sustained Ca^2+^ signals, not only in the acinar cells, but also in the normal quiescent stellate cells. These Ca^2+^ signals cause both acinar and stellate cell necrosis.^5^ When most of the stellate cells are dead, chronic pancreatitis cannot develop because only these cells are capable of producing the fibrotic microenvironment. With regard to alcohol-related pancreatitis, it turns out that *in vivo*, but not in isolated cells, alcohol and fatty acids promote the transition of quiescent to activated stellate cells, although the mechanism responsible for this has not yet been clarified.^6^ Alcohol and fatty acids, generating fatty acid ethyl esters inside the cells, elicit large Ca^2+^ signals in both acinar and quiescent stellate cells,^6^ but in the activated stellate cells, the Ca^2+^ signals elicited by this combination are very much smaller than in the quiescent cells, preventing necrosis.^6^ Why do the activated stellate cells generate much smaller, and therefore less toxic, Ca^2+^ signals in response to alcohol and fatty acids than the quiescent ones? Kusiak et al. show that the principal cause of the diminished Ca^2+^ signals is downregulation of the TRPA1 channel. This reduces Ca^2+^ influx and thereby protects against the development of toxic Ca^2+^ signals that overload the mitochondria with Ca^2+^ and reduce ATP generation. This protection makes the activated stellate cells markedly resistant to cell death. In alcohol-related acute pancreatitis, the stellate cells therefore largely survive and in their activated state they can produce the fibrotic microenvironment that is potentially cancer promoting (Figure 1).

**Figure 1.**
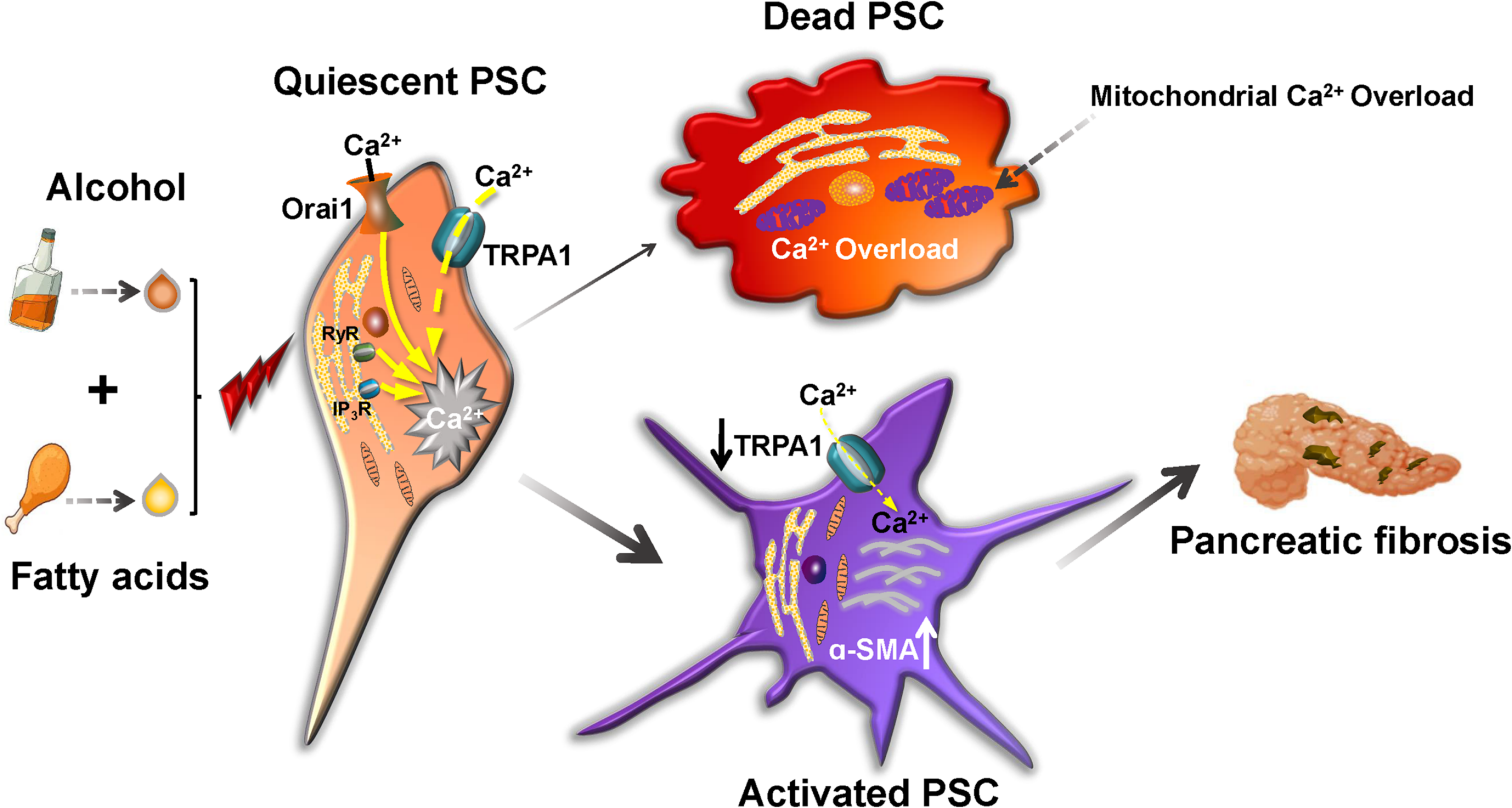
Schematic diagram illustrating that downregulation of TRPA1 in activated pancreatic stellate cells prevents the toxic Ca^2+^ signal generation that results in necrosis of quiescent stellate cells (see text for further explanation).

The new results also indicate that TRPA1 plays an important role in the Ca^2+^ signal generation evoked by alcohol and fatty acids in the quiescent stellate cells. It has previously been shown that BK-elicited opening of TRPA1 channels depends on phospholipase C activation.^3^ Kusiak et al. now show that Ca^2+^ signal generation in the stellate cells depends on primary release of Ca^2+^ from the endoplasmic reticulum, almost certainly mediated via IP_3_ receptors, which are expressed in the stellate cells.^6^ However, the precise mechanism by which alcohol and fatty acids cause opening of TRPA1 channels is unknown. The toxic Ca^2+^ signals elicited by alcohol and fatty acids in the pancreatic acinar cells have also been shown to depend on primary Ca^2+^ release from the endoplasmic reticulum, mostly through IP_3_ receptors but, in order to develop a sustained elevation of the cytosolic Ca^2+^ concentration, opening of Ca^2+^ release activated Ca^2+^ channels of the Orai1 type is required.^5^ The toxic effects of alcohol and fatty acids on the acinar cells can be prevented by pharmacologically blocking the Orai1 channels.^5^ The BK-elicited Ca^2+^ signals in stellate cells have also been shown to depend on Orai1 channel activity.^5^ It is therefore somewhat confusing that the Ca^2+^ signals generated by alcohol and fatty acids in the stellate cells would seem to depend on TRPA1 channels rather than Orai1 channels,^6^ since in both cases the primary event is release of Ca^2+^ from the endoplasmic reticulum.^5,6^ In this context, it is a difficulty that in the new study of Kusiak et al., all Ca^2+^ signaling experiments were carried out on cultured human stellate cells, whereas previous work^5^ was done on stellate cells in their normal environment in the exocrine mouse pancreatic tissue. Cultured cells may have different properties from normal cells in their normal environment, but species differences can also not be excluded. Specifically with regard to TRPA1 channels, it is interesting that caffeine has been shown to activate mouse TRPA1 channels, whereas it suppresses human TRPA1 channels.^9^

Although it is now accepted that both stellate cells and macrophages play a crucial role in the development of acute pancreatitis, the actual initiation of the disease process has generally been attributed to the toxic Ca^2+^ signals generated in the acinar cells by the combination of alcohol and fatty acids or by bile acids. These signals cause acinar necrosis and release of proteases, including trypsin and kallikrein, which in turn, via various intermediary steps, generate Ca^2+^ signals in stellate cells and macrophages thereby amplifying the inflammatory processes.^5^ The new results presented by Kusiak et al., showing that alcohol and fatty acids can also directly elicit Ca^2+^ signals in the stellate cells,^6^ raise the intriguing possibility that acute pancreatitis may be initiated by simultaneously occurring Ca^2+^ signaling events in both acinar and stellate cells. It may be important to note that the SARS-CoV-2 spike protein has very recently been shown to elicit Ca^2+^ signals primarily in the stellate cells. This leads secondarily to Ca^2+^ signal generation in adjacent macrophages, most likely due to cytokine secretion from the stellate cells.^10^ Mounting evidence therefore now indicates that the stellate cells play a more direct role in the initiation of acute pancreatitis than originally thought.

Although activation of TRPA1 is generally accepted to be proinflammatory,^1–3^ the data presented by Kusiak et al. indicate that there can be situations in which inhibition of TRPA1 channel function paradoxically turns out to be proinflammatory.^6^ Clearly extensions of the work of Kusiak et al. to pathophysiological situations *in vivo* will be important in order to determine the potential general importance of this new concept.

## Data Availability

There are no data presented in this perspective.
